# Prostatic epithelial cells and their high expressions of CKIP-1 affect the TGF-β_1_ expression levels which might reduce the scar formation in remodeling stage at prostatic urethral wounds after wound repair

**DOI:** 10.1007/s11255-019-02286-z

**Published:** 2019-09-21

**Authors:** Lixin Wang, Ying Cao, Zhizhong Guan, Guangheng Luo, Lei Luo, Xiushu Yang, Mingliang Chu

**Affiliations:** 1 Department of Pathology, Guizhou Provincial People’s Hospital, Guiyang, 550002 Guizhou China; 2 Department of Pathology, Guizhou Medical University, Guiyang, 550002 Guizhou China; 3 Department of Urology, Guizhou Provincial People’s Hospital, Guiyang, 550002 Guizhou China

**Keywords:** Wound healing, Prostatic urethra wound, Less scar, TGF-β_1_, CKIP-1

## Abstract

**Objective:**

There are less scar formations in some wounds after wound repair. Our earlier study had shown that the amount of collagen fibers in canine prostatic urethra wound were less than in bladder neck wound after 2-μm laser resection of the prostate (TmLRP) and partial bladder neck mucosa at 4 weeks. The purpose of this study was to observe the amount of scar tissue and characterize the probable causes of “less scar healing” in prostatic urethra wound.

**Methods:**

A total of 12 healthy adult male crossbred canines underwent resection of prostate and partial bladder neck mucosa using 2-μm laser. The prostatic urethra and bladder neck wound specimens were harvested at 3, 4, 8 and 12 weeks after operation, respectively. The histopathologic characteristics were observed by hematoxylin and eosin(HE)staining, and the expression of transforming growth factor-β_1_ (TGF-β_1_) and casein kinase-2 interacting protein-1 (CKIP-1) were examined by immunohistochemistry in prostatic urethra and bladder neck wound, respectively. Overexpressed CKIP-1 human prostate epithelial cells (BPH-1 cells) were established and the expression of TGF-β_1_ was detected by Western blotting. Furthermore, a non-contact co-culture system of BPH-1 cells and human fibroblast (HFF-1) cells was used to observe the effects of BPH-1 cell and their high CKIP-1 levels on the expression of TGF-β_1_ in HFF-1 in vitro.

**Results:**

The histology showed that there were a large number of prostatic epithelium and a small amount of scar tissue in prostatic urethra wound, while no epithelial cells and more scar tissue in bladder neck wound at 4, 8 and 12 weeks after repair. There were a higher expression level of TGF-β_1_ in prostate epithelial cells and fibroblasts and a lower expression level of CKIP-1 in prostate epithelial cells at 3 weeks after surgery in prostatic urethral wound. Compared to week 3, the TGF-β_1_ expression decreased both in prostate epithelial cells and fibroblasts at 4, 8 and 12 weeks in prostatic urethral wound (*p* < 0.05 or *p* < 0.01). The CKIP-1 expression increased in prostate epithelial cells at 4, 8 and 12 weeks compared to 3 weeks in prostatic urethra wound (*p* < 0.01). A higher TGF-β1 expression level of fibroblasts was observed in bladder neck wound at 3 weeks. And there was no significant change in the expression of TGF-β_1_ of fibroblasts in 3, 4, 8 and 12 weeks after operation in bladder neck wound. Both the prostate urethra and bladder neck wound fibroblasts showed weak expression of CKIP-1 and there was no significant change in 3, 4, 8 and 12 weeks. The vitro experiments showed that the TGF-β_1_ expression in BPH-1 cells with CKIP-1 overexpression decreased 25% compared with control group (*p* < 0.05). Furthermore, the expression of TGF-β_1_ in HFF-1 cells of co-cultured group decreased by 20% compared with Control group (*p* < 0.05); the expression of TGF-β_1_ in HFF-1 cells of overexpression co-culture group were reduced by 15% compared with co-cultured group (*p* < 0.01).

**Conclusions:**

A large number of prostate epithelial cells in prostatic urethra wound may be one of the causes of less formation of scar tissue after repair. The prostate epithelial cells might reduce expression level of TGF-β_1_ by raising CKIP-1 expression and inhibit expression of TGF-β_1_ in peripheral fibroblasts at remodeling stage to reduce the excessive proliferation of fibrous cells and the excessive scar formation.

## Introduction

In the wound healing process, the wound often forms more scar tissue which seriously influences physical and mental health of the patient. Nevertheless, the exact mechanism of scar formation remains unclear. Now, studies focus on how to avoid or reduce scar formation in the wound repair. Recently, studies found that in certain wounds there only were no or a small amount of scar tissue, and this type of wound repair was called “scarless or less scar healing”. For example, Burrington and his team [1] investigated the wound healing of embryonic skin and found that fetal wounds heal without scarring. In addition, compared with skin wounds, the oral mucosa is less scarred after wound repair [2], the incision in the buccal mucosa causes scar formation and incision in the gums but no obvious scar formation [3]. This phenomenon arouse people’s interest in exploring these wounds “scarless or less scar healing” mechanisms. Benign prostate hyperplasia (BPH) is one of the most common diseases affecting aging males. Approximately 20% of all BPH patients with symptomatic disease eventually undergo surgery [4]. After surgery, the wound repair mechanism immediately activates to repair the tissue defect induced by operation injury. In our previous studies of prostatic urethra wound healing, we found that re-epithelialization of prostatic urethra wound occurs faster, when re-epithelialization is completed, the expression of collagen fibers is relatively low compared with the bladder neck wound in the same canine at 3 and 4 weeks after surgery [5, 6]. Clinical cases of bladder neck contracture (BNC) after operation are often reported in patients with BPH [7]. It is believed that the bladder neck is caused by excessive electrical burning during the operation and the contracture caused by scar hyperplasia, nonetheless postoperative prostatic urethral wound scar hyperplasia contracture has been rarely reported in the existing literature. Less scar tissue that is produced after the repair of prostatic urethra wound aroused our interest in the study of the prostatic urethral wound repair mechanism, since it can help us to find out the possible causes of inhibiting the formation of wound scar and the ideas for prevention of scars.

Wound healing is a complex process involving multiple cellular components, cytokines, and network synergy between extracellular matrixes [8]. Transforming growth factor-β_1_ (TGF-β_1_) is one of the important cytokines in wound healing process, and it is also the most closely growth factors related to scar formation [9]. It believed that the low TGF-β_1_ expression in the later stage of wound healing process is one of the reasons of scarless or low amount of scar [3, 10]. Nevertheless, the exact mechanism that reduces or terminates the TGF-β_1_ expression in the later stage of wound healing remains unclear. Over the recent years, it has been suggested that casein kinase 2 interaction protein 1 (CKIP-1) may be the negative regulatory factor of TGF-β_1_ and may play a regulatory role in wound healing [11, 12], providing a new idea for us to learn how to reduce the expression of TGF-β_1_ in the late stage of prostatic urethra wound. In this current study, a canine two-micron (2 μm) laser prostate and bladder neck resection model was established. The histopathologic characteristics, the expression of TGF-β_1_ and CKIP-1 were observed by hematoxylin and eosin (HE) and immunohistochemistry staining in prostatic urethra and bladder neck wound at 3, 4, 8, and 12 weeks after treatment, respectively. In the cell model, the possible causes of less scar wound healing in prostatic urethra wound were discussed. The results might provide new ideas and data to understand the mechanism of less scar healing in prostatic urethra wound.

## Materials and methods

### Animals

Twelve healthy adult male crossbred canines, 5–7 years old, weighing 18–22 kg were obtained from Zunyi Medical College. All the animals were housed in an environment with temperature of 22 ± 1 °C, relative humidity of 50 ± 1% and a light/dark cycle of 12/12 h. All animal studies (including the euthanasia procedure) were done in compliance with the regulations and guidelines of Guizhou Provincial People’s Hospital institutional animal care and conducted according to the AAALAC and the IACUC guidelines.

### Modeling of 2-μm laser resection of prostate and partial bladder neck mucosa

All operations were performed using the same 2-μm continuous wave Tm:YAG laser system (RevoLix; Lisa Laser Products, Katlenburg, Germany). The laser wavelength was 2.013 mm and the energy was transmitted at 70 W of power output through flexible 550-mm-diameter fiber. Briefly, animals were anesthetized using 10% chloral hydrate (0.003 ml g^−1^) and then placed in the supine position on the operating table. The lower abdomen was entered through a medial and longitudinal incision, and the anterior wall of the bladder was freed. A purse suture was performed in the anterior wall of the bladder. Consequently, the incision was made within the purse to allow the placement of a 26F continuous-flow resectoscope, and the suture was firmly fixed. Under saline irrigation, a resectoscope was placed into the prostatic urethra through the internal urethral orifice. The prostatic laser vaporesection procedure was performed as previously described [13]. Each animal also underwent the resection of partial bladder neck mucosa using 2-μm laser simultaneously with the prostatic vaporesection at considerable distance from the prostatic urethra wound. After completing the procedure, the bladder and the abdominal wall were closed. In addition, no transurethral catheter was required.

### Histopathologic examination

The animals were randomly divided into four groups (3 canines/group). The canines were killed, and wound specimens from prostatic urethra wound and bladder neck wound were harvested and fixed in 4% formalin at 3, 4, 8, and 12 weeks after laser treatment. Prostate tissue samples were cut in the transverse plane at the level of the mid-prostatic urethra to permit inspection of the lesion. After embedding in paraffin, 4-μm slides were examined histologically by HE staining.

### Immunohistochemistry staining

Immunohistochemical staining was performed as described earlier [14]. Briefly, the sections were treated with blocking buffer (Dako Denmark A/S, Glostrup, Denmark) for 30 min at room temperature (RT) and thereafter incubated with anti-TGF-β_1_ antibody (1:200 dilution in Tris–NaCl buffer; Abcam, USA) and anti-CKIP-1 antibody (1:500 dilution in Tris–NaCl buffer; Abcam, USA) at 4 °C overnight. After rinsing in Tris–NaCl buffer, the sections were incubated with biotinylated goat anti-rabbit IgG (diluted 1:200 in Tris–NaCl buffer) for 60 min at RT. Sections were subsequently incubated with avidin-biotinylated enzyme complex and DAB and then dehydrated with increasing concentrations of ethanol, cleared with xylene, and mounted in Permount. Negative controls for these immunohistochemical procedures were incubated with nonimmune serum instead of the primary antibodies, which resulted in no detectable staining.

The optical densities (ODs) of TGF-β_1_ and CKIP-1-like immunoreactivity (IR) in prostatic urethra and bladder neck wound at 3, 4, 8 and 12 weeks after surgery were measured using a CM2000B Biomedical Image Analysis System (Beihang University, China). The OD of TGF-β_1_ and CKIP-1 were analyzed by microdensitometry in prostatic epithelial cells and fibroblast cells at prostatic urethra and bladder neck wound, respectively. Five random fields of interest were measured, and the OD measurements were averaged.

### Cell lines

Human prostate epithelial cell line (BPH-1) and human fibroblast cell line (HFF-1) were obtained from American Type Culture Collection (ATCC, VA, USA). Cells were cultured in DMEM (Hyclone, USA) containing 10% fetal calf serum in a humidified atmosphere containing 5% CO_2_/95% air at 37 °C.

### Plasmids, lentiviral production and infection–construction of CKIP-1 overexpressing BPH-1 cells line

The pLenti CMV/TO Puro empty vector was acquired from Addgene (Addgene plasmid#17482 [15]). The genomic segment of CKIP-1 sequences was amplified using primers CKIP-1-F:5′-ATGATGAAGAAGAACAATTCCG-3′ and CKIP-1-R:5′-TCACATCAGGCTCTTCCGGTAC-3′ and then subcloned into BamH1 and Xbal sites of the pLenti CMV/TO Puro empty vector [16]. And then, we divided the contents of the experiment into three groups, namely: blank control group, negative control group (also known as the empty plasmid pLenti CMV group) and CKIP-1 overexpression group (also known as pLenti CMV-CKIP-1 transfection group). Lentivirus production and transduction were done according to a previous method [16].

### Non-contact co-culture of cells

A non-contact co-culture system of BPH-1 and HFF-1 cells was established using a Transwell suspension culture chamber with polyethylene terephthalate film combined with a six-pore plate (Corning 3450; Corning, Inc., Corning, NY, USA). Cells were divided into three groups: control group, co-culture group and overexpression co-culture group. Control group, contained HFF-1 cells alone in the transwell system (HFF-1 cell in the lower chamber and no cell in the upper chamber). Co-culture group, contained BPH-1 cells and HFF-1 cells, were co-cultured in the transwell system (BPH-1 in the upper chamber and HFF-1 cells in the lower chamber). Overexpression co-culture group, contained CKIP-1 overexpression BPH-1 cells and HFF-1 cells, were co-cultured in the transwell system (CKIP-1 overexpression BPH-1 cell in the upper chamber and HFF-1 cells in the lower chamber). The number of cells seeded per chamber for each group was 5 × 10^4^ cells. Cells were cultured in six-well plates (Corning 3450) containing the aforementioned complete medium of 5% CO_2_. They were cultured in an incubator at a constant temperature of 37 °C to extract cell protein after 72 h.

### Western blotting

The cells were washed twice with PBS and resuspended in cold RIPA buffer containing 1 mmol/L phenylmethanesulfonyl fluoride and a cocktail of protease inhibitors (dilution, 1:100; Beyotime, Nantong, China). The cell samples were centrifuged at 12,000 rpm at 4 °C for 15 min. Supernatants were recovered and total proteins were quantified using BCA Protein Assay kit (Beyotime). Total protein was extracted and separated by 10% SDS-polyacrylamide gel electrophoresis and then transferred onto the PVDF membranes (Millipore, USA). After being blocked for 2 h at room temperature, the membranes were incubated with 1:1000 dilution of polyclonal anti-rabbit CKIP-1 (Abcam, USA), 1:500 dilution of polyclonal anti-TGF-β_1_ antibody (Abcam, USA) and 1:2000 dilution of polyclonal GADPH (Abcam, USA) overnight. The proteins were then incubated with the corresponding secondary antibody for 1 h at room temperature. After washing, PVDF membranes were transferred into the Bio-RadChemiDoc TM XRS system. The signals were captured, the intensity of the bands was analyzed by using the Image Lab^TM^ Software (Bio-Rad, USA) and normalized to the corresponding signal for GADPH. Finally, the relative level of each protein is expressed as a percentage of the level (as 100%) of that protein in control group.

### Statistical analysis

Data were expressed as the mean ± standard deviation. SPSS v. 22.0 software (SPSS, Inc., Chicago, IL, USA) was used to determine statistical significance between groups. For statistical comparisons, parametric analysis of variance with the Turkey’s test or one-way anova was done. *p* < 0.05 indicated a statistically significant difference.

## Results

### Histopathology changes of canine prostatic urethra and bladder neck wound after 2-μm laser prostate and bladder neck resection at 3, 4, 8 and 12 weeks

**Fig. 1:**
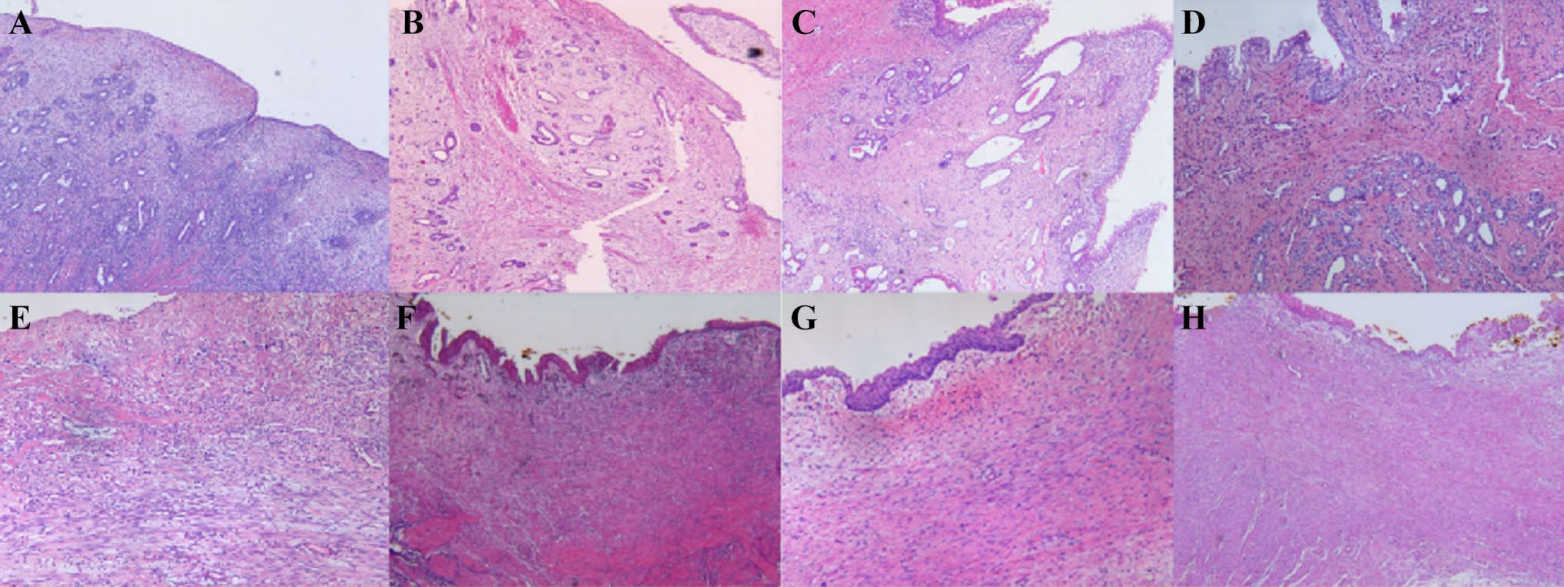
Histopathological changes of canine prostatic urethra and bladder neck wounds after 2-μm laser prostate and bladder neck resection. At 3, 4, 8 and 12 weeks (× 200). **a** At 3 weeks of prostatic urethra wounds, the wound was covered by regeneration epithelium. There are lots of prostate epithelium inside the wound, the granulation tissue was gradually mature. **b** At 4 weeks of prostatic urethra wounds, there are lots of prostate epithelium inside the wound, and the granulation tissue was more mature, inflammation cells and capillaries were reduced, and slight fibrous tissue hyperplasia was observed. **c** At 8 weeks of prostatic urethra wounds, there were many prostate acinar and ductal epithelium in the wound. The granulation tissue around the epithelium transformed into fibrous tissue with mild hyperplasia of fibrous tissue. **d** At 12 weeks of prostatic urethra wounds, there are lots of prostate epithelium in the wound. Fibrous tissue showed mild hyperplasia and a small amount of scar formation. **e** At 3 weeks of bladder neck wounds, below the wound surface, granulation tissue was gradually replaced by fibroblasts. **f** At 4 weeks of bladder neck wounds, the surface of bladder neck wound was largely covered with new epithelium. Granulation tissue gradually becoming fibrotic and obvious fibrous tissue hyperplasia was observed. **g** At 8 weeks of bladder neck wounds, large number of parallel collagen fibers, slender fibrous cells, and obvious hyperplasia were observed within the wound area. **h** At 12 weeks of bladder neck wounds, fibrous tissue significantly hyperplasia and formation

Three weeks after operation in canine prostatic urethra wound, the prostatic urethra wound was covered by regeneration epithelium which had polar arrangement and umbrella cell on its surface. There are lots of prostate epithelium inside the wound, the granulation tissue around these prostate epithelial cells was gradually mature (Fig. 1a). At 4 weeks after surgery, histopathology changes were basically the same as 3 weeks. Nevertheless, the granulation tissue was more mature, inflammation cells and capillaries were reduced, and slight fibrous tissue hyperplasia was observed (Fig. 1b). At 8 weeks of prostatic urethra wound, urothelium was covered on all wound surfaces. There were many prostate acinar and ductal epithelium in the wound. The granulation tissue around the epithelium was mature and transformed into fibrous tissue with mild hyperplasia of fibrous tissue (Fig. 1c). At 12 weeks of canine prostatic urethra wound after resection, there are lots of prostate epithelium in the wound. Fibrous tissue showed mild hyperplasia and a small amount of scar formation (Fig. 1d).

**Fig. 2:**
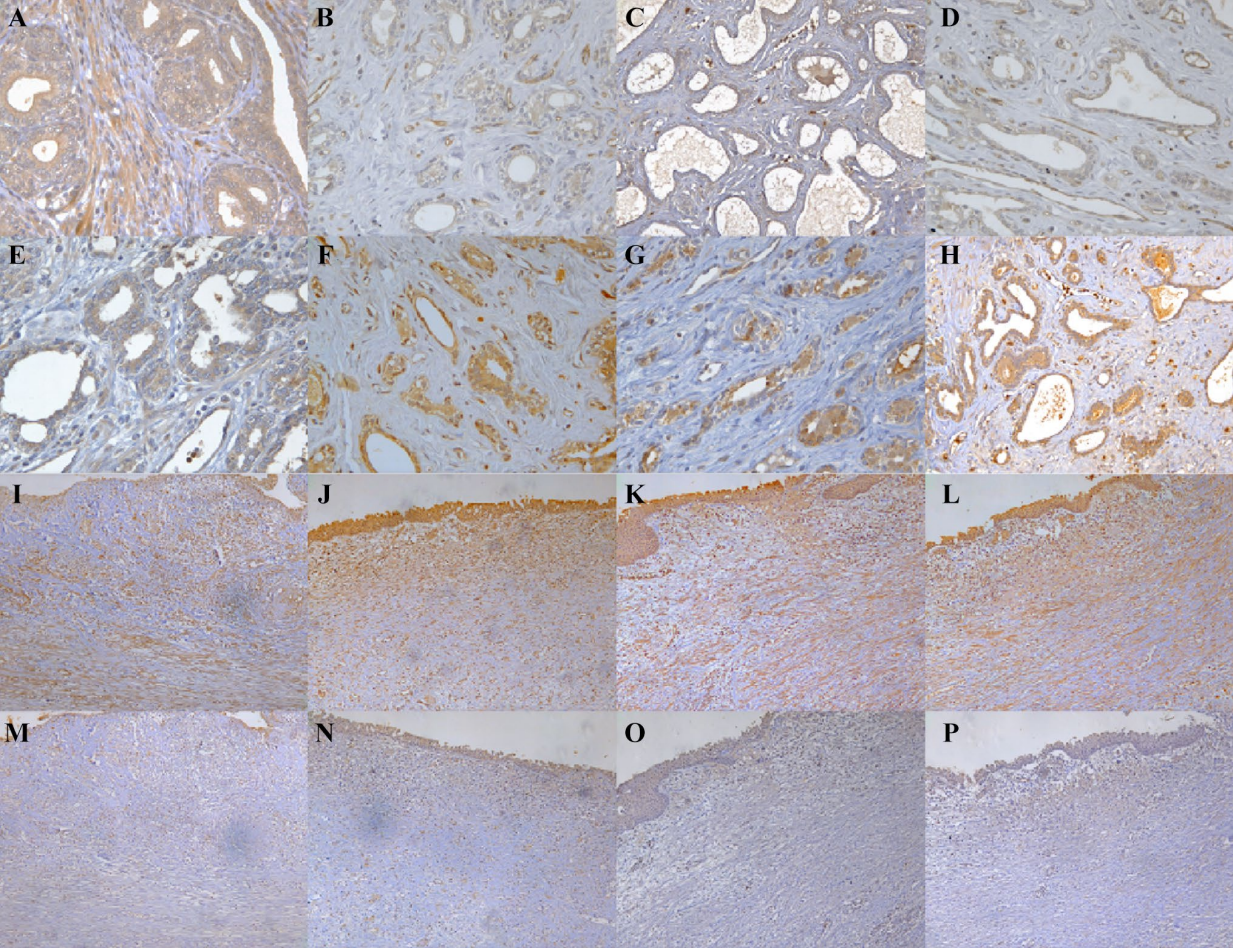
The expression of TGF-β_1_ and CKIP-1 in canine prostatic urethra and bladder neck wounds at 3, 4, 8 and 12 weeks after 2-μm laser prostate and bladder neck resection (Envision × 200). TGF-β_1_ expression at **a** 3 weeks, **b** 4 weeks, **c** 8 weeks and **d** 12 weeks after surgery in prostatic urethra wound; CKIP-1 expression at **e** 3 weeks, **f** 4 weeks, **g** 8 weeks and **h** 12 weeks after surgery in bladder neck wound

At 3 weeks in canine bladder neck wound, there were distinct proliferating epithelial cells in the edges of the wound at the bladder neck. Epithelial regeneration commenced from the edges of the bladder neck wound and partly wounds were covered by regenerated epithelium. Below the wound surface, granulation tissue was gradually replaced by fibroblasts (Fig. 1e). At 4 weeks after operation, the surface of bladder neck wound was largely covered with new epithelium. But, there were still few wounds areas without regeneration epithelium. Granulation tissue gradually becoming fibrotic and obvious fibrous tissue hyperplasia was observed compared to prostate urethral wound (Fig. 2f). At 8 weeks, large number of parallel collagen fibers, slender fibrous cells, and obvious hyperplasia were observed within the wound area (Fig. 2g). Twelve weeks in canine bladder neck wound, fibrous tissue significantly hyperplasia and formation (Fig. 2h).

### The TGF-β_1_ and CKIP-1 expression at canine prostatic urethra and bladder neck wound after 2-μm laser prostate and bladder neck resection at 3, 4, 8 and 12 weeks

Positive expression of TGF-β_1_ in cytoplasm was observed in inflammatory cells, fibroblasts, regeneration urothelial cells and prostate epithelial cells at canine prostatic urethra wound. Significantly higher expression of TGF-β_1_ was observed in prostate epithelial cells and fibroblasts at the 3rd week (Fig. 2a) compared to 4th, 8th and 12th week (Fig. 2b–d) (*p* < 0.05 or *p* < 0.01). In addition, the prostate epithelial cells and regenerated urothelial cells have shown to be positive for CKIP-1 at canine prostatic urethra wound, while it was weakly expressed in the fibroblasts. The expression of CKIP-1 was strikingly increased in the prostate epithelial cells at week 4, 8 and 12 compared to week 3, but the CKIP-1 expressed of fibroblasts were no significant difference over time (Fig. 2e–h) (*p* < 0.01).

In canine bladder neck wound, TGF-β_1_ expression was found in both inflammatory cells, fibroblasts and regenerated urothelial epithelium. In the fibroblasts, significantly higher expression of TGF-β_1_ was observed. However there were no significant difference in the expression of TGF-β_1_ over time (Fig. 4i–l). In addition, the weak positive expression of CKIP-1 in fibroblasts were seen and no strikingly differences were observed at 3, 4, 8 and 12 weeks (Fig. 4m–p). The average OD of TGF-β_1_ and CKIP-1 in canine prostatic urethral and bladder neck wound at 3, 4, 8 and 12 weeks after resection of prostate and bladder neck are shown in Table 1.

**Table 1:** The average OD of TGF-β_1_ and CKIP-1 in canine prostatic urethra and bladder neck wounds at 3, 4, 8 and 12 weeks after 2-μm laser prostate and bladder neck resection ($\overline{x}$ ± S)

	Prostatic urethra wound				Bladder neck wound	
	TGF-β_1_		CKIP-1		TGF-β_1_	CKIP-1
	Prostatic epithelial cells	Fibroblast	Prostatic epithelial cells	Fibroblast	Fibroblast	Fibroblast
3 weeks	0.22 ± 0.02	0.23 ± 0.02	0.14 ± 0.02	0.07 ± 0.01	0.23 ± 0.03	0.09 ± 0.01
4 weeks	0.15 ± 0.01*	0.13 ± 0.01^△^	0.26 ± .01**	0.08 ± 0.02	0.24 ± 0.02	0.08 ± 0.02
8 weeks	0.14 ± 0.02**	0.14 ± 0.03^△^	0.25 ± .02**	0.08 ± 0.03	0.25 ± 0.02	0.06 ± 0.01
12 weeks	0.13 ± 0.02**	0.13 ± 0.02^△^	0.23 ± .03**	0.06 ± 0.02	0.22 ± 0.03	0.06 ± 0.02

Data are presented as mean ± SD. *p < 0.05 or **p < 0.01 vs prostate epithelial cells in 3 weeks; ^△^*p* < 0.01 vs fibroblast in 3 weeks

### Expression of TGF-β_1_ in CKIP-1 overexpression BPH-1 cells

**Fig. 3:**
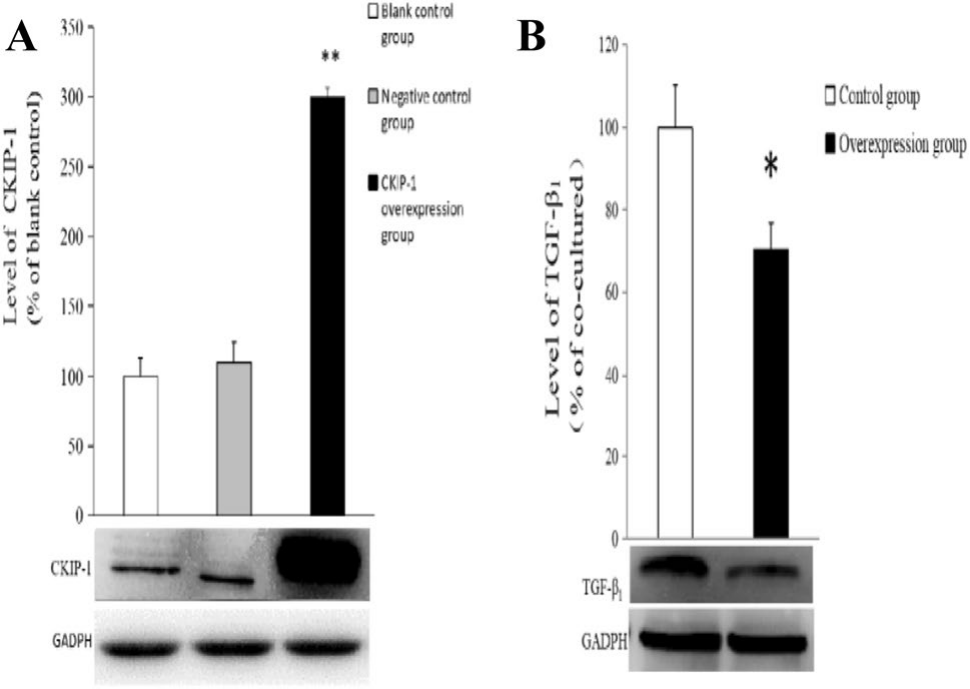
The expressions of CKIP-1 (**a**) and TGF-β_1_ (**b**) in CKIP-1 overexpression BPH-1 cells. Data are presented as mean ± SD. *p < 0.05 or **p < 0.01 vs control group and negative control group

The expression of CKIP-1 in three groups of BPH-1 cell (blank control group, the empty plasmid pLenti CMV group and pLenti CMV-CKIP-1 transfection group) were detected by Western blotting, respectively. Compared to the blank control group and the empty plasmid pLenti CMV group, the expression of CKIP-1 in CKIP-1 overexpression group is significant increase (*p* < 0.01) (Fig. 3a), suggesting the CKIP-1 overexpression BPH-1 cells model is success. Next, we examined the TGF-β_1_ expression in CKIP-1 overexpression BPH-1 cells. The result showed that the expression level of TGF-β_1_ of BPH-1 cells in CKIP-1 overexpression group was reduced by 25% compared with the control group (*p* < 0.05) (Fig. 3b).

### The TGF-β_1_ expression of HFF-1 cells after co-culture with BPH-1 cells or CKIP-1 overexpression BPH-1 cells

**Fig. 4:**
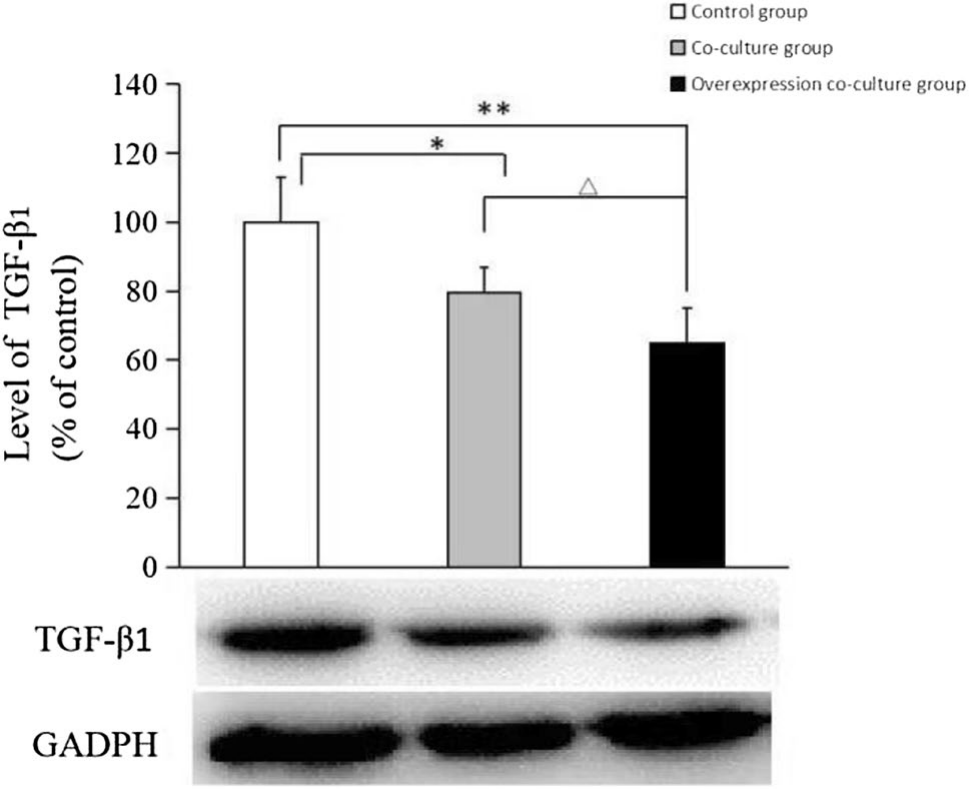
The expression of TGF-β_1_ in HFF-1 cells after co-culture with BPH-1 cells or CKIP-1 overexpression BPH-1 cells. Data are presented as mean ± SD. *p < 0.05, **p < 0.01 vs control group; ^△^*p* < 0.05 vs co-culture group

Compared to the control group, the expression of TGF-β_1_ in HFF-1 cells of co-culture group decreased by 20% (*p* < 0.05) and in HFF-1 cells of CKIP-1 overexpression co-culture group decreased by 35% (*p* < 0.01). Further analysis showed the expression of TGF-β_1_ decreased by 15% in CKIP-1 overexpression co-culture group than in co-culture group (*p* < 0.05). The expressions of TGF-β_1_ in three groups of HFF-1 cells are shown in Fig. 4.

## Discussion

Wound healing is a defensive adaptive response of the organism, which repairs the local tissue defect caused by disease or injury and restores the structure and function of the wound to the greatest possible extent. For a long time, wound healing brings some troubles to clinicians. On the one hand, the wound surface is difficult to heal due to various reasons. On the other hand, the wound is healed by pathological means such as scar hyperplasia and scar contracture. Trauma repair includes tissue regeneration, granulation tissue hyperplasia, maturation and scar tissue formation, and is usually divided into inflammatory, hyperplasia and remodeling stage consisting of three periods. Inflammation stage mainly includes hemostatic and inflammatory response; proliferative stage mainly consists of the wound granulation tissue hyperplasia that fills the tissue defect and re-epithelialization to close the wound; during remodeling stage, granulation tissue gradually matures, and fibroblasts gradually transforms into fibrous tissue and scar tissue. Therefore, scar is the inevitable result of tissue repair when the trauma reaches a certain level. Scar tissue can repair the wound to maintain the integrity and firmness of the tissue and organ. However, a large amount of scar tissue in the wound will affect the normal structure and function, and the quality of life of the patient.

At present, the exact mechanism of scar formation is not very clear, and numerous studies focus on improving the quality of wound healing to reduce wound scar tissue formation. Recently, studies have shown that only a small amount of scar tissue is formed in certain wounds, providing a new perspective to understand scar formation. In our previous studies [5, 6] of wound healing in canine prostatic urethra wound, we found that at 4 weeks after surgery, when re-epithelialization was complete, there was less fibrous tissue in prostatic urethra wound as compared to bladder neck wound in the same canine. In clinical practice, scar hyperplastic contracture of the prostatic urethra wound in BPH patients after surgery are rarely reported. This phenomenon prompted us to focus the possible cause of less scar formation in prostate urethral wound. In this current study we established 2-μm laser resection of canine prostate and partial bladder neck mucosa animal models, and extended the animal model time to 8 and 12 weeks (remodeling stage of wound healing) to further observe the amount of scar tissue and compare the difference of histopathologic characteristics in this two wounds. Briefly, our results still showed an obvious hyperplasia of fibrous and a large amount of scarring in bladder neck wound, while slight hyperplasia of fibrous tissue and a small amount of scarring in prostate urethra wound were observed at 8 and 12 weeks after surgery, which means the prostate urethra wound repair might be “less scar healing”. We observed and compared the histopathologic differences in these two wounds and found that there are a large number of prostate epithelial cells in prostate urethral wound after repair, and no epithelial cells in bladder neck wound. This phenomenon is associated with the difference of re-epithelialization source cells in this two wound. Our preliminary histopathologic studies [5, 6] have shown that the re-epithelialization of the canine bladder neck wound resulted from the mobilization of proliferating transitional epithelial cells from the edges of the bladder neck wound analogous to skin wound repair. However, the re-epithelialization of prostatic urethra wound results from the migration and differentiation of proliferating epithelium from the residual acinar and ductal prostatic epithelium under the wound, not from the transitional epithelium at the edges of the bladder neck, a clinical concept that has traditionally prevailed among urologists. Why our organism selected residual prostatic epithelium under the wound but not the transitional epithelium at the edges of the bladder neck to complete re-epithelialization in prostatic urethra wound? We consider there may be two possible reasons. Firstly, prostatectomy has only resected the hyperplasia of the prostate gland, the prostate membrane is not removed, so there are still a large number of prostate epithelial tissue under the wound. Secondly, the prostate cells might have more remarkable proliferative, migration and differentiation capacity [17] than transitional epithelial cells to achieve re-epithelialization in a short time interval. Thus, the re-epithelialization from the bottom–up prostatic cells lead to the presence of a large number of prostate epithelial cells in the prostate urethral wounds after completing of the restoration of the wound.

Numerous studies have suggested that epithelial cells in the wound might regulate the scar tissue formation. In the wound healing process of third-degree skin burns, granulation tissue gradually becomes fibrotic, resulting in great scar tissue. However, in second-degree skin burns in which dermal appendages have not been lost, there is limited fibrous and scar tissue. People realize that certain tissues in the dermis, such as skin attachment tissue, might have some inherent characteristics to prevent the development of fibrous and scar tissue formation in wound healing process [18]. Studies on tissue engineering of urethral repair also show that, when urethral damage is large, urethra wound were easy to occur urethral stricture and fibrosis when urethra wound was repaired with pure decellularized stromal [19]. While the urethra was repaired with the stroma inoculated with epithelial cells, no stricture occurs in the reconstruction of urethra [20]. According to the above studies, we speculate the causes of “less scar healing” in prostatic urethra wound, part of the reasons may be that the rapid re-epithelialization result in the wound can be closed as soon as possible to form less granulation tissue in the wound, so the collagen fibers and scar tissue after the granulation tissue maturation are less. Another important cause may be the large number of prostate epithelial cells in prostatic urethra wound might secrete certain substances to reduce the generation of collagen fibers or provide protection against scar formation.

Cytokines have a very important role in the defense and repair mechanisms following trauma. Among them, TGF-β_1_ is strongly involved in wound healing process. Many tissues and cells can secrete TGF-β_1_, which then affects the surrounding cells by self-secretion or side-secretion. In inflammatory and hyperplasia stage of skin wound repair, platelets, inflammatory cells, keratinocyte and fibroblasts all can secrete TGF-β_1_ to promote local inflammatory reaction, accelerate the proliferation of wound granulation tissue and re-establish the epithelium. Yet, the overexpression of TGF-β_1_ at remodeling stage of wound healing can lead to the continuous proliferation of fibroblasts in the wound, which may induce excessive hyperplasia and the formation of scar [9, 21]. At present, the lower TGF-β_1_ expression level in remodeling stage of repair is considered one of the important reasons for scar-free and lower scar formation in embryonic skin and oral mucosa wound [3, 10]. Nevertheless, the exact mechanism of how to reduce the TGF-β1 expression during remodeling stage in these wounds still remains unclear. In recent years, the discovery of CKIP-1 might provide new ideas to learn how our organism reduces the expression TGF-β1 in remodeling stage of wound repair. CKIP-1 is a pleckstrin homology domain-containing protein that was originally identified as an interacting protein of casein kinase-2 α-subunit (CK2α) [22]. CKIP-1 has been involved in various biological functions, including megakaryocytic differentiation, chronic heart failure, cardiac hypertrophy, cancer cell proliferation and apoptosis, and osteoporosis [23–27]. Recent studies suggested that CKIP-1 may be a negative regulator of TGF-β1 and might play an importance regulatory role in wound repair. TGF-β1/Smad signaling pathway is directly associated with scar formation [28]. Smads protein is an important downstream regulatory protein in TGF-β1 signaling [29] and Smad ubiquitination regulatory factor 1 (Smurf1) is the first cytokines to be found to degrade Smads. Zhang et al. [12] have found that CKIP-1 is an important regulatory factor of Smurf1, which can enhance the activity of Smurf1 to inhibit the Smads expression and then regulate the expression of TGF-β1.

In order to observe whether there is expression change of TGF-β1 and CKIP-1 in prostatic urethra and bladder neck wound, we detected the TGF-β1 and CKIP-1 expression in 3, 4, 8 and 12 weeks by immunohistochemical staining. Our data showed there were a higher expression level of TGF-β1 in prostate epithelial cells and fibroblasts in prostatic urethral wound at 3 weeks (hyperplasia stage) after surgery. When the re-epithelialization was complete, the expression of TGF-β1 decreased both in prostate epithelial cells and fibroblasts in prostatic urethra wound at remodeling stage (4, 8 and 12 weeks) compared to week 3. We also observed a higher TGF-β1 expression level of fibroblasts in bladder neck wound at 3 weeks. However, there were no significant lower of TGF-β1 expression at 4, 8 and 12 weeks compared to week 3 in bladder neck wound. In inflammatory and hyperplasia stage of wound repair, TGF-β1 can be secreted from different cells (such as platelets, inflammatory cells, epithelial cells and fibroblasts) to promote inflammatory reaction, accelerate the proliferation of granulation tissue and re-epithelialization. In remodeling stage, the platelet and inflammatory cells in the wound were significantly reduced, epithelial cells and fibroblasts from granulation tissues in the wound were the main secreted sources of TGF-β1. Our result showed a lower TGF-β1 expression both in prostate epithelial cells and fibroblasts in prostatic urethra wound at remodeling stage, which might be one of the causes of prostatic urethra wound “less scar healing”. Furthermore, our immunohistochemical results showed that the CKIP-1 expression of prostatic epithelial cells increased at 4 weeks and lasted 8 and 12 weeks compared with 3 weeks after surgery in prostatic urethra wound. While the CKIP-1 expression of fibroblasts were weakly both in prostatic urethra and bladder neck wound, and no striking differences were observed in 3, 4, 8 and 12 weeks. Based on this above results, we speculate that prostate epithelial cells might up-regulation the CKIP-1 expression to reduce TGF-β1 secretion at remodeling stage of repair in prostatic urethra wound. However, we did not observe the CKIP-1 expression in fibroblasts increased in remodeling stage in prostatic urethra wound. So, the low TGF-β1 expression level of fibroblasts was not caused by the up-regulation of CKIP-1 expression of fibroblasts. Then, to survey whether the low TGF-β1 expression level in fibroblasts is related to surrounding prostate epithelial cells and their higher CKIP-1 expression, we explored these problems in vitro experiments. First, we established CKIP-1 overexpressing BPH-1 cells line and detected the expression of TGF-β1 by Western blotting. As expected, lower TGF-β1 expression was found in CKIP-1 overexpression BPH-1 cells compared to the control cells. This suggested that prostate epithelial cells may up-regulation the CKIP-1 expression to reduce TGF-β1 secretion. Furthermore, we established non-contact co-culture model of HFF-1 cell and BPH-1 cell (or CKIP-1 overexpression BPH-1 cell), and surveyed the TGF-β1 expression of HFF-1 cell after 72 h of co-culture. Our data showed that the TGF-β1 expression in HFF-1 cells of co-culture group decreased compared with Control group; the TGF-β1 expression in HFF-1 cells of Overexpression co-culture group were reduced compared with co-cultured group. These results suggested that the TGF-β1 expression of HFF-1 cells was lower when it was co-culture with BPH-1 cell, and was further lower when it was co-culture with CKIP-1 overexpression BPH-1 cells. Combined with co-culture experimental results, we consider that the lower TGF-β1 level of fibroblasts at remodeling stage in prostatic urethra wound is related to surrounding prostate epithelial cells and their higher CKIP-1 expression level. We speculate the prostate epithelial cells could inhibit surrounding fibroblasts TGF-β1 expression, and up-regulated the CKIP-1 expression level of prostate epithelial cells could promote this effect. Since CKIP-1 is not a secretory protein and cannot act on adjacent target cells in a paracrine manner. How do the prostate epithelial cells with high CKIP-1 expression to affect the TGF-β1 secretion of surrounding fibroblasts? We speculate whether prostate epithelial cells down-regulates its own TGF-β1 expression by up-regulation of CKIP-1, resulting in a low level of TGF-β1 environment around fibroblasts, which inhibits fibroblasts TGF-β1 expression. In addition, CKIP-1 can interact with a variety of cellular proteins and affect multiple signal transduction pathways. Whether CKIP affects other secretory cytokines expression to affects the TGF-β1 expression of fibroblasts through paracrine needs further research.

Our experiment provides some ideas for how to promote the wound repair and reduce the wound of scar formation. First, the repair process was derived from the residual prostatic tissue after prostatectomy, and a large number of prostatic acinar and ductal epithelial cells appeared in the wound during the process. This may indicate the cells from prostatic acinar and ducts play an important role during the wound healing process. To explore more about those may be a good idea to know the mechanism of the wound healing process. BPH patients with resection of the prostate, just resect the hyperplasia of prostate tissue to open channel, don’t injury or resection of the prostate membrane to ensure there are enough prostate epithelial cells in wound repair. In the second place, to some easy-to-form scar wounds, it is advisable to inoculate epithelial cells and up-regulate the expression of CKIP-1 to reduce the formation of scar tissue. However, our study only found some phenomena, but the exact mechanism needs further study and discussion. There are still many deficiencies in our study. We only focused on the effect of prostate epithelial cells on fibroblasts, and did not consider whether fibroblasts would have an effect on prostate epithelial cells. In the cell experiment we used non-contact co-culture cell model, and whether the two kinds of cells inhibited proliferation and secretion by contact inhibition was not considered. No discuss whether the cause of “less scar healing” of prostate urethral wounds was related to the excessive degradation of extracellular matrix. These above questions all need to be discussed in the future research.
